# Extraction of Metal Ions with Metal–Organic Frameworks

**DOI:** 10.3390/molecules24244605

**Published:** 2019-12-16

**Authors:** Natalia Manousi, Dimitrios A. Giannakoudakis, Erwin Rosenberg, George A. Zachariadis

**Affiliations:** 1 Laboratory of Analytical Chemistry, Department of Chemistry, Aristotle University of Thessaloniki, 54124 Thessaloniki, Greece; 2 Institute of Physical Chemistry, Polish Academy of Sciences, Kasprzaka 44/52, 01-224 Warsaw, Poland; DAGchem@gmail.com; 3 Institute of Chemical Technology and Analytics, Vienna University of Technology, 1060 Vienna, Austria; egon.rosenberg@tuwien.ac.at

**Keywords:** MOFs, metals, extraction, sample preparation, microextraction, spectrometry, environmental samples, food samples, biological samples

## Abstract

Metal–organic frameworks (MOFs) are crystalline porous materials composed of metal ions or clusters coordinated with organic linkers. Due to their extraordinary properties such as high porosity with homogeneous and tunable in size pores/cages, as well as high thermal and chemical stability, MOFs have gained attention in diverse analytical applications. MOFs have been coupled with a wide variety of extraction techniques including solid-phase extraction (SPE), dispersive solid-phase extraction (d-SPE), and magnetic solid-phase extraction (MSPE) for the extraction and preconcentration of metal ions from complex matrices. The low concentration levels of metal ions in real samples including food samples, environmental samples, and biological samples, as well as the increased number of potentially interfering ions, make the determination of trace levels of metal ions still challenging. A wide variety of MOF materials have been employed for the extraction of metals from sample matrices prior to their determination with spectrometric techniques.

## 1. Introduction

The terminology of metal–organic frameworks (MOFs) was initially introduced in 1995, when Yaghi and Li reported the synthesis of a new “zeolite-like” crystalline structure upon the polymeric coordination of Cu ions with 4,4′-bipyridine and nitrate ions, resulting to large rectangular channels [1]. MOFs are known to have superior characteristics, such as high surface area (theoretically up to 14.600 m^2^g^−1^) [2], porosity of uniform in structure and topology nanoscaled cavities, and satisfactory thermal and mechanical stability. Therefore, metal–organic frameworks were established as successful candidates for various applications like environmental remediation, detoxification media of toxic vapors, heterogeneous catalysis, gas storage, imaging and drug delivery, fuel cells, supercapacitors, and sensors [2,3,4,5,6,7,8,9,10,11,12,13].

In the field of analytical chemistry, MOFs have been employed in various analytical sample preparation methods including solid-phase extraction (SPE), dispersive solid-phase extraction (d-SPE), magnetic solid-phase extraction (MSPE), stir bar sorptive extraction (SBSE), and pipette tip solid-phase extraction (PT-SPE) [14,15,16,17,18]. Metal–organic frameworks have been also tested as stationary phases for high-performance liquid chromatography (HPLC), capillary electrochromatography (CEC), and gas chromatography (GC) with many advantages. Moreover, with the use of chiral MOFs, separation of chiral compounds has been also reported [19,20,21,22].

**Figure 1:**
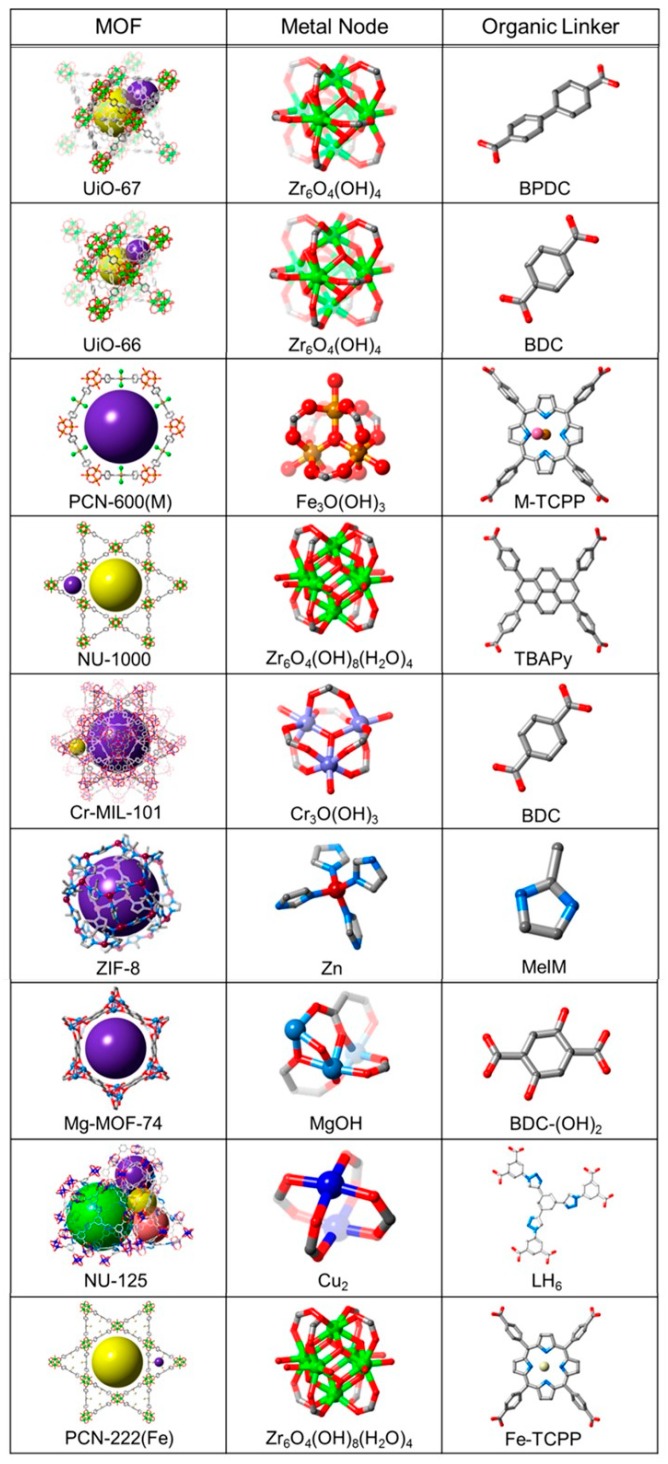
Examples of Metal–Organic Frameworks. Adapted with permission from Reference [25]. Copyright (2016) American Chemical Society.

Metal–organic frameworks have been synthesized and successfully applied for the preconcentration of heavy metals from environmental samples prior to their detection/analysis with a spectroscopic technique. The most common metal ions used in MOFs are Zn(II), Cu(II), Fe(III), and Zr(IV), while terephthalic acid, trimesic acid, or 2-methylimidazole have been excessively used as organic linkers [23]. Many efforts have been made in order to overcome the low water stability of MOFs toward the preparation of suitable sorbents for the extraction of metal ions [24]. Examples of MOFs are presented in Figure 1 [25]. Compared with other sorbent materials, MOFs have a significant advantage of stable and homogeneous pores of specific sizes [26].

The effect of trace heavy metals on human health has attracted worldwide attention. Their increasing industrial, domestic, agricultural, and technological utilization has resulted in wide distribution in the environment. Metals such as cadmium, lead, mercury, chromium, and arsenic are considered as systemic toxicants and it, therefore, is essential to determine their levels in environmental samples [27]. Among the different analytical techniques that are widely used for the determination of metal ions are flame atomic absorption spectroscopy (FAAS), electrothermal atomic absorption spectroscopy (ETAAS), inductively coupled plasma optical emission spectrometry (ICP-OES), and inductively coupled plasma mass spectrometry (ICP-MS) [28,29,30].

Due to the low concentrations of metals and the presence of various interfering ions in complex matrices, the direct determination of such ions at trace levels is still challenging. Various novel materials including graphene oxide, activated carbon, carbon nanotubes, porous oxides, and metal–organic frameworks have been successfully employed for this purpose [31,32,33,34].

Until now, a plethora of articles discuss the perspective of the use of MOFs in analytical chemistry [19,20,24,26,34,35,36,37,38]. Most of the reported review articles are focused on the extraction of organic compounds from food, biological, and environmental matrices. Herein, we aim to discuss the applications of MOFs as potential sorbents for the extraction of metal ions prior to their determination from environmental, biological, and food samples. Application of subfamilies of MOFs, such as zeolitic imidazole frameworks (ZIFs) or covalent organic frameworks (COFs), will also be discussed.

## 2. Stability of MOFs in Aquatic Environment

The stability of the framework in aqueous solutions depends on the strength of the metal–ligand coordination bonds [39]. The collapse of MOFs in the presence of water is linked to the competitive coordination of water and the organic linkers with the metal ions/nodes. The stability of the structure is also associated with other factors like the geometry of the coordination between metal-ligand, the surface hydrophobicity, the crystallinity, and the presence of defective sites [40]. The use of additives like graphite oxide, graphitic carbon nitride, nanoparticles, or the deposition on substrates such as carbon, fibers, or textiles, can have a positive effect on the framework stability [41,42,43,44,45,46,47]. In order to evaluate the stability and as a result the properness of utilizing a MOF for adsorption application, the pH and the temperature under which the preconcentration of the metal will take place, must be considered.

The strength of the coordination between the organic moieties and the metal ions can be described in general according to the HSAB (hard/soft acid/base) principles [9,47]. Zr^4+^, Fe^3+^, Cr^3+^, and Al^3+^ are regarded as hard acidic metal ions, while Cu^2+^, Zn^2+^, Ni^2+^, Mn^2+^, and Ag^+^ as soft ones [39]. On the other hand, carboxylate-based linkers act as hard bases, while azolate ligands (such as pyrazolates, triazolates, or imidazolates) as soft bases. For that reason, most of the Zr-based UiO (University of Oslo) and MIL-53(Fe) (Material Institut Lavoisier) series possess remarkable water stability, while for instance one of the most known and studied MOF, HKUST-1 (Hong Kong University of Science and Technology) does not. On representative paradigm of Zn-based water-stable structure is the zeolitic imidazolate framework (ZIF), formed from imidazolate ligands and Zn^2+^.

When used in analytical chemistry, MOFs must be stable both under adsorption and under desorption conditions. Usually, adsorption of metal ions takes place under weakly acidic conditions (pH = 5–6), while desorption is performed predominately with the addition of a strong acid. However, even though many MOFs are stable under adsorption conditions, they are decomposed with the addition of strong acids like nitric, hydrochloric, and sulfuric acid [24,29]. Other reagents that have been employed for the elution of metal ions without decomposing the MOF material are ethylenediaminetetraacetic acid (EDTA), sodium chloride (NaCl), or sodium hydroxide (NaOH) solution in EDTA or in thiourea.

## 3. Mechanisms of Metal Ions Extraction with Metal–Organic Frameworks

**Figure 2:**
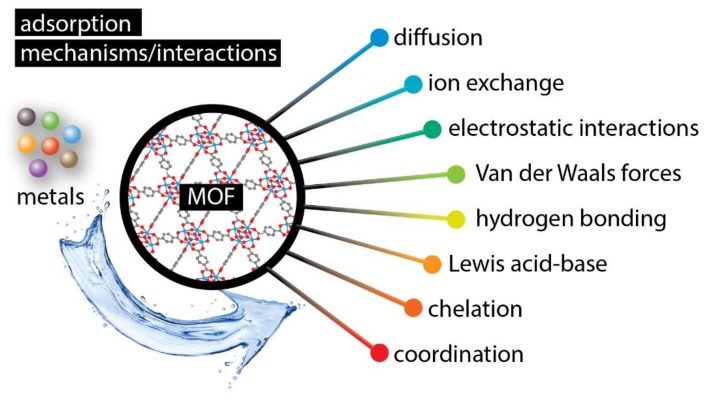
A schematic illustration of the interactions/mechanisms involved in the adsorption of metals by metal–organic frameworks (MOFs).

MOFs, as well as their composites, have been successfully applied as adsorbents for various heavy metal/metalloid species. The adsorption of the latter from aquatic environments is still among the ultimate research targets, and there are plenty of reports in which adsorption/removal of heavy metals was a success story [48,49,50]. Although, not all MOFs are water-stable as discussed above. The most widely reported interactions/mechanisms are collected in Figure 2 [51]. In many cases, more than one mechanism is responsible for the high adsorptive capability of MOFs. The binding/interaction sites can be either the metal or the clusters as well as the linkers. In order to enhance the adsorptive capability and/or selectivity, the functionalization of the linkers, with groups as hydroxyl, thiol, or amide, is a well-explored and successive strategy.

Lewis acid–base interactions are the most common adsorption mechanism of metal ions by metal–organic frameworks [52]. The presence of O-, S-, and N-containing groups that act as Lewis bases is very important for the preconcentration of the various ionic species from aqueous solution since metal ions act as Lewis acids. The donor atoms of the MOFs are present in the molecules of the organic linkers. Pre- or post-synthesis functionalization of the frameworks can increase the number of O-, S-, or N-containing groups in order to enhance the adsorption selectivity and efficiency of the target metal ions. Since Lewis acid–base interactions are critical for metal adsorption onto the donor atoms of the MOFs, it is obvious that the pH of the solution plays the most critical role, influencing the adsorption process and kinetics. In low pH value, those atoms are protonated, and adsorption cannot take place due to the repulsive forces of the cationic form of metal with the positively charged adsorption sites [53]. However, by increasing the pH of the aqueous samples that contain the metal ions, the donor atoms of the adsorbent are deprotonated and they become favorable for complex formation and sorption of the target analytes. In basic solutions, the addition of hydroxide may lead to complex formation and precipitation of many metals, therefore, after a certain pH value, any further increase can lead to a decrease of the sorption efficiency [54,55].

Adsorption by coordination is another adsorption mechanism in which the functionalization plays a key role. For instance, Liu et al. showed that the post-synthetic modification of Cr-MIL-101 with incorporation of -SH functionalities led to an improvement of Hg(II) removal, even at ultra-low concentrations [56]. This improvement was linked to the coordination between Hg(II) with the -SH groups. The incorporation of thiol-containing benzene-1,4-dicarboxylic acid (BDC) linkers in the case of UiO-66 MOF resulted in a material capable of simultaneously adsorbing As(III) and As(V) oxyanions. The adsorption of the former occurred via coordination to the -SH groups, while of the latter by the binding of the oxyanions to the Zr_6_O_4_(OH)_4_ cluster via hydroxyl exchange [57]. The hydroxyl exchange mechanism was also proposed as the predominant capturing pathway in the study of Howard and co-workers [58], in which they studied the adsorption of Se(IV) and Se(VI) in water by seven Zr-based MOFs (UiO-66, UiO-66-NH_2_, UiO-66-(NH_2_)_2_, UiO-66-(OH)_2_, UiO-67, NU-1000, and NU-1000BA).

Additionally, the adsorption mechanism with metal–organic frameworks can be enhanced via the chelation mechanism, after functionalization of MOFs with compounds that can form chelating complexes with the metal ions [59]. For example, functionalization of metal–organic frameworks with dithizone can enhance Pb extraction by forming penta-heterocycle chelating complex compounds. In this case, the binding sites of the chelating molecules are also protonated in low pH values and adsorption cannot take place. Adsorption capacity increases with increasing pH until a certain point, normally at a pH value of 5 to 6. Further increase in pH value can lead to precipitation of the target analytes, due to hydrolysis [60].

In the case of the physical-based adsorption, various interactions can be responsible for the elevated adsorptive capability of MOFs as mentioned above. The net charge of the framework and the presence of specific functional groups have a positive impact on the extent of the physical interactions [61]. The manipulation of the above can be achieved by grafting of particular species/groups into the framework or by tuning the net charge as a result of the solution pH in which the adsorption takes place.

The electrostatic interactions between the negatively charged adsorption sites of MOFs with the oppositely charged adsorbates are the most widely reported pathway [62]. The diffusion of the metal ions toward the active sites prior to the blockage of the outer entrances of the channels is also an important aspect and so, the volume, geometry, and size of the pores are of paramount importance [63].

## 4. Sample Preparation Techniques for the Extraction of Metal Ions

Solid-phase extraction (SPE) is a well-established analytical technique that has been widely used for the extraction, preconcentration, clean-up, and class fractionation of various pollutants from environmental, biological, and food samples. Different sorbents have been evaluated for the SPE procedure usually placed into cartridges [64]. MOFs have been employed as sorbents for the solid-phase extraction. In a typical SPE application, the sorbent is conditioned to increase the effective surface area and to minimize potential interferences, prior to the loading of the sample solution onto a solid-phase [65,66,67]. The analytes are retained onto the active sites of the sorbent and the undesired components are washed out. Finally, elution of the analytes with the desired solvent is carried out [54].

**Figure 3:**
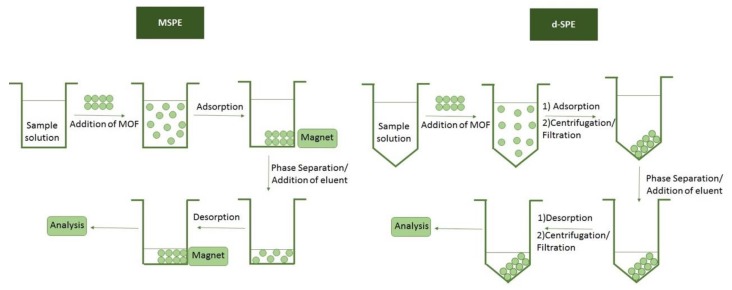
Typical magnetic solid-phase extraction (MSPE) and dispersive solid-phase extraction (d-SPE) procedures for the enrichment and analysis of trace metal ions.

SPE and other conventional sample preparation techniques like protein precipitation and liquid–liquid extraction (LLE) have fundamental drawbacks such as time-consuming complex steps, difficulty in automation, and need for large amounts of sample and organic solvents. Novel extraction techniques, including MSPE, d-SPE, SBSE, and PT-SPE, have been developed in order to overcome these problems. Figure 3 shows the typical steps of MSPE and d-SPE. Recently, MOFs have been used as sorbents for these extraction techniques [68].

Dispersive solid-phase extraction is performed by direct addition of the sorbent into the solution that contains the target analytes. Various MOF materials have been employed for the d-SPE of metal ions from complex sample matrices. After a certain time, the sorbent is retrieved from the solution with centrifugation or filtration and the solution is discarded. Elution with an appropriate solvent is performed and the liquid phase is isolated for instrumental analysis. The dispersion is often enhanced by stirring, vortex mixing, or ultrasound irradiation, in order to enable an efficient transfer of the target analytes to the active sites of the sorbent. Therefore, several devices including shakers, vortex mixers, and ultrasonic probes and baths have been implemented for sorbent dispersion. Until today, the ultrasound-assisted dispersive solid-phase microextraction is the most common d-SPE approach [24,69].

MSPE is based on the use of sorbents with magnetic properties. There are several different procedures to fabricate magnetic MOFs that have been employed to prepare sorbents for MSPE. The most common approaches are the direct post-synthesis of magnetic MOF materials with magnetic nanoparticles and the second one, in situ growth of magnetic nanoparticles during the synthesis of the framework. In the first case, the desired MOF and the magnetic nanoparticles (Fe_3_O_4_) are synthesized separately and mixed under sonication. For the in situ approach, the MOF is added to a solution containing the reagents for the synthesis of Fe_3_O_4_ in order to give a magnetic material. Moreover, single-step MOF coating can take place by adding the Fe_3_O_4_ nanoparticles into a mixture of inorganic and organic precursors for MOF synthesis. Carbonization of some MOFs can shape magnetic nanoparticles due to aggregation of the metallic component of the MOF. At the same time, the organic linker is converted to a porous carbon. Finally, the layer-by-layer approach is based on the sequential immobilization of the different components of the MOFs into a functionalized support.

For the typical MSPE procedure, a magnetic sorbent is added to the sample for sufficient time in order to ensure a quantitative extraction. After this period of time, an external magnet is employed to retrieve the sorbent and the sample is discarded. The sorbent is washed and an appropriate solvent is added in order to desorb the analytes. After magnetic separation, the eluent can be directly analyzed or it can be evaporated and reconstitute in an appropriate solvent prior to the analysis [70,71].

Other extraction techniques that can be coupled with MOFs in order to extract different analytes from complex matrices are stir bar sorptive extraction (SBSE) and pipette tip solid-phase extraction (PT-SPE). SBSE is an equilibrium technique, initially introduced by Baltussen et al. In this technique, extraction of the analytes takes place onto the surface of a coated stir bar [72,73,74]. PT-SPE is a miniaturized form of SPE in which ordinary pipette tips act as the extracting column and small amount of sorbent is packed inside the tip [75,76]. Only a small range of SBSE and PT-SPE sorbents are commercially available, which limits the possible applications of those techniques. MOF materials have been successfully used as coatings for stir bars and as packed sorbents in pipette tips [72,73,74,75,76].

Although MOFs pose several benefits as extraction sorbents for SPE, MSPE d-SPE, SBSE, and PT-SPE, their water stability and selectivity have to be enhanced with appropriate functional groups or pore functionalization. Therefore, the type of metal–organic framework and the possible functionalization should be carefully chosen. Other parameters that should be thoroughly investigated are the pH value of the sample solution, the extraction and desorption time, the desorption solvent, etc.

As mentioned before, the pH of the sample solution is one of the most critical parameters for the extraction of heavy metals from aqueous samples. Therefore, the pH value has to be optimized carefully in order to allow the Lewis acid–base interactions between the sorbent and the target analytes and to prevent precipitation due to hydrolysis.

The mass of the MOF material, as well as the extraction time, are other parameters that can influence the extraction step and require optimization. First of all, an optimum adsorbent amount is necessary in order to maximize the extraction efficiency. Certain extraction time is also required to facilitate the interaction between the analytes and adsorption sites of the MOF material. Finally, the sample volume and the volume of the eluent has to be optimized in order to provide a higher enrichment factor that is possible.

Regarding the desorption step, among the parameters that should be thoroughly investigated are the type, the volume, and the concentration of the eluent. In most cases, elution can be achieved with acidic solutions of nitric or hydrochloric acid. The presence of H^+^ ions weakens the interaction between the analyte and the MOF, as it competes for binding with the active sites of the adsorbent. However, decomposition of most MOFs has been observed in acidic conditions. Other reagents that have been used for the elution of metal ions without decomposing the MOF material are EDTA, NaCl, NaOH in EDTA, NaOH in thiourea, etc. Furthermore, enough desorption time should be provided in order to enable the quantitative elution of the adsorbed analytes.

Other parameters that can be investigated are the stirring speed, salt addition, the use of ultrasonic radiation, etc., depending on the extraction procedure [74,75,76,77,78]. The optimization of the experimental parameters can be performed by evaluating one-factor-at-a-time or by performing Design of Experiments (DoE), such as Box–Behnken experimental design [79].

Finally, the effect of potentially interfering ions that naturally occur in the various sample matrices, the adsorption capacity of the MOF material, as well as the reusability of the sorbent should be also evaluated [74,75,76,77,78].

## 5. Applications of Metal–Organic Frameworks for the Extraction of Metal Ions

The applications of MOFs for the extraction of metal ions from environmental, biological, and food samples, as well as the obtained recoveries and limits of detection (LODs), are summarized in Table 1.

### 5.1. Extraction of Palladium

In 2012, Bagheri et al. [80] synthesized a MOF material using trimesic acid and copper nitrate trihydrate. The metal–organic framework was modified with pyridine functionalized Fe_3_O_4_ (Fe_3_O_4_@Py) nanoparticles and used for the preconcentration of Pd (II) from aqueous samples prior to its determination by FAAS. Modification with pyridine was performed to increase selectivity toward palladium. Optimization of extraction and elution steps was performed with the Box–Behnken experimental design through response surface methodology [79]. The developed method was used for the analysis of fish, sediment, soil, tap water, river water, distilled water, and mineral water. Acid digestion with nitric acid (for fish samples) and nitric acid with hydrochloric acid (for soil and sediment) was carried out prior to the MSPE procedure. The researchers observed that hydrochloric acid and nitric acid decomposed the structure of the magnetic MOF sorbent; however, 0.01 mol L^−1^ NaOH in potassium sulfate provided quantitative recovery without any decomposition. The developed method showed high sample clean-up as well as satisfactory recovery values and enhancement factors [79].

### 5.2. Extraction of Lead

Lead(II) has been extracted from water samples with the implementation of a metal–organic framework sustained by a nanosized Ag12 cuboctahedral node [65]. The MOF material was prepared from silver nitrate, melamine, and malonic acid. The sorbent was packed in a glass column and secured with polypropylene frits. For the extraction, the sample was loaded onto the column and lead was desorbed with EDTA prior to its determination by FAAS. Due to the cage-like structure of the MOF material and the presence of melamine and malonic acid, rapid and selective adsorption of lead was achieved resulting in a SPE method with low LODs, high extraction recoveries, and good enhancement factors. No significant decrease in binding affinity was observed for the repeated use of the sorbent (up to five times).

A dithizone-functionalized magnetic metal–organic framework was synthesized by Wang et al. and applied for the magnetic solid-phase extraction of lead from environmental water samples prior to its determination by ETAAS [81]. For the synthesis of the material, a Fe_3_O_4_ functionalized copper benzene-1,3,5-tricarboxylate was further functionalized with dithizone (DHz). The dithizone functionalized MOF exhibited good adsorption efficiency and selectivity toward lead via chelation mechanism. Elution was performed with 2.0 mol L^−1^ HNO_3_ and even though nitric acid is known to decompose many MOF materials, the prepared sorbent was found to be reusable for at least 80 times under acidic condition. Furthermore, with the use of the developed MSPE sorbent, a rapid, reliable and highly selective method for lead quantification was developed.

Lead has been also extracted from food samples with metal–organic framework adsorbent modified with mercapto groups prior to determination by FAAS [77]. The MOF material was prepared from copper nitrate trihydrate and trimesic acid and was subsequently modified with Fe_3_O_4_ nanoparticles functionalized with mercapto groups (Fe_3_O_4_@SH). Elution was performed with 1 mol L^–1^ of HNO_3_, however, no sorbent reusability or data about sorbent decomposition was reported. The developed MSPE method was successfully applied for the analysis of rice, pig liver, tea, and water samples. The presence of thiol groups in combination with the high surface area of the sorbent enhanced significantly the sensitivity of the determination.

Finally, lead has been determined in cereal, beverages, and water samples, using the highly porous zirconium-based MOF-545 [82]. The novel sorbent was implemented for the vortex-assisted d-SPE of lead prior to determination by FAAS. The material was prepared from zirconyl chloride octahydrate and meso-tetra(4-carboxyphenyl) porphyrin in dimethylformamide (DMF). Prior to the extraction procedure, cereals, legumes, and juices (chickpeas, beans, wheat, lentils, and cherry juice) were dried and digested with nitric acid and hydrogen peroxide while mineral water was used without digestion. High adsorption capacity achieved as well as low LOD values. The sorbent demonstrated good stability after the elution with 1 mol L^−1^ HCl and was found to be reusable for up to 42 times.

### 5.3. Extraction of Mercury

Mercury has been extracted from fish samples with HKUST-1 prior to its determination of Hg(II) using cold vapor atomic absorption spectroscopy (CVAAS) [83]. The MOF material was prepared from trimesic acid and copper acetate and was subsequently modified with Fe_3_O_4_ nanoparticles functionalized with 4-(5)-imidazole-dithiocarboxylic acid. After elution of mercury with 0.01 mol L^−l^ thiourea solution, the sorbent was found to be reusable for up to 12 times. This novel method was used for the extraction of mercury from fish and canned tuna samples providing low LODs as well as satisfactory recovery values.

A porous metal–organic framework was prepared from thiol-modified silica nanoparticles and copper complex of trimesic acid and used for the extraction of Hg(II) from water and fish samples [78]. For this purpose, SH@SiO_2_ nanoparticles were prepared from SiO_2_ and (3-mercaptopropyl)-trimethoxysilane. The thiol-modified nanoparticles were mixed with trimesic acid and copper acetate monohydrate in a DMF/ethanol solution to give the desired MOF sorbent. The optimum elution solvent was found to be 0.01 mol L^−1^ NaOH since it provided satisfactory recoveries without structure decomposition. The copper benzene-1,3,5-tricarboxylate sorbent was used for the d-SPE of Hg(II) from tap, river, sea and wastewater, fish, and sediment samples prior to cold vapor atomic absorption spectrometry. The developed method was simple, selective, rapid, low-cost, environment- friendly and provided high enrichment factor. Although the preparation of MOF material was complicated, large quantity of sorbent can be prepared at once.

Mercury was also extracted from tea and mushroom samples with a JUC-62, prepared from 3,3′5,5′-azobenzenetetracarboxylic acid and copper nitrate trihydrate. [84] Tea samples were dried and digested with nitric acid prior to the d-SPE procedure. The novel sorbent was studied in both static and kinetic adsorption mode, and the static mode showed excellent adsorption capacity. Acetate buffer (0.02 M, pH 4.6) was chosen for elution and the sorbent was found to be reusable for up to 3 times. Mercury was finally measured by atomic fluorescence spectrometry (AFS).

A mesoporous porphyrinic zirconium metal–organic framework (PCN-222/MOF-545) was synthesized and used for the pipette-tip solid-phase extraction of Hg ions from fish samples prior to their determination by cold vapor atomic absorption spectrometry [76]. For the preparation of the MOF, 200 mg of zirconyl chloride octahydrate, benzoic acid, and meso-tetrakis(4-carboxyphenyl)porphyrin were used. For the extraction procedure, two milligrams of the sorbent were placed into a pipette-tip and 1.8 mL of the sample were aspirated and dispensed into a tube for 10 repeated cycles, while elution was performed with 15 μL of hydrochloric acids (10% *v*/*v*) at 15 cycles. The total analysis time was less than 7 min, the novel MOF material could be used for at least 15 extractions–desorption cycles without any change in its extraction efficiency and the preconcentration method provided 120-fold enhancement for mercury.

### 5.4. Extraction of Copper

In 2014, Wang et al. [29] synthesized a superparamagnetic Fe_3_O_4_-functionalized metal–organic framework from Fe_3_O_4_ nanoparticles zinc nitrate hexahydrate and 2-aminoterephthalic acid in DMF with the hydrothermal approach. The reaction mixture was heated to 110 °C for 24 h in a Teflon liner. The obtained IRMOF-3 material was used to determine Cu(II) ions by electrothermal atomic absorption spectrometry. Sulfuric, nitric, and hydrochloric acids were found to decompose the sorbent, therefore, 0.1 mol L^−1^ NaCl solution (pH = 2) was used for the elution of the adsorbed analytes. After optimization of the extraction procedure, the novel sorbent was successfully applied for the analysis of tap and lake water. The novel sorbent was found to be reusable for at least 10 times without any significant decrease in recovery. Due to the presence of abundant amine groups in the MOF material, high adsorption capacity and extraction efficiency toward the target analyte was achieved.

### 5.5. Extraction of Cadmium

Cadmium (II) ions have been preconcentrated from environmental water samples with a sulfonated MOF loaded onto iron oxide nanoparticles (Fe_3_O_4_@MOF235(Fe)-OSO_3_H) [85]. For the synthesis of the sorbent, mercaptoacetic acid functionalized Fe_3_O_4_ (Fe_3_O_4_@MAA) nanoparticles were mixed with terephthalic acid and iron chloride hexahydrate in DMF. The reaction mixture was placed into an autoclave and heated at 85 °C for 24 h. Finally, the sulphonated MOF loaded onto the magnetic nanoparticles was prepared by the suspension of Fe_3_O_4_@MOF-235(Fe) in aminomethanesulfonic acid (AMSA). A solution of 0.5 mol L^−1^ EDTA was used to elute the adsorbed analyte. The obtained functionalized MOF material exhibited good stability, reusability (up to 10 times), as well as low toxicity. The novel sorbent was used for MSPE of cadmium prior to FAAS determination and enhancement factor of 195 was achieved. The Langmuir isotherm indicated that cadmium was adsorbed as the monolayer on the homogenous adsorbent surface.

### 5.6. Extraction of Thorium

UiO-66-OH metal–organic framework has been successfully applied for the selective d-SPE and trace determination of thorium from water samples prior to its determination by spectrophotometry [86]. The MOF material was prepared from zinc chloride and 2-hydroxyterephthalic acid in DMF after heating at 80 °C for 12 h. The developed method showed high extraction efficiency and capacity toward Th after its chelating with morin. The developed metal–organic framework exhibited low toxicity, reusability for more than 25 times (after elution with 0.2 mol L^−1^ HNO_3_) as well as high stability. The d-SPE method showed high accuracy, low LODs, and high tolerance to co-existing ions.

Thorium has been also monitored in natural water with a dual-emission luminescent europium organic framework. The MOF material was synthesized from europium(III) acetate hexahydrate and [1,1′-biphenyl]-4-carboxylic acid in DMF with a solvothermal approach. After thorium uptake, the emission spectrum of the metal–organic framework was excited by UV irradiation. The LOD of the reported procedure was found to be 24.2 μg L^−1^ [87].

### 5.7. Extraction of Uranium

Uranium has been extracted from natural water samples with a hydrolytically stable mesoporous terbium(III)-based luminescent mesoporous MOF equipped with abundant Lewis basic sites [88]. High sensitivity and selectivity were achieved in real lake samples, where there is a huge excess of potentially interfering ions. The MOF material was prepared from terbium nitrate hexahydrate and 4,4′,4″-(1,3,5-triazine-2,4,6-triyltriimino)tris-benzoic acid in DMF. The reaction mixture was at 100 °C for 72 h into a Teflon-lined reactor. Desorption of uranium was performed with nitric acid and the sorbent was found to be stable under acidic conditions since it was found to be reusable for at least 3 times. The novel sorbent was successfully used for the d-SPE of uranyl ions prior to its determination by ICP-MS. Uranium uptake by MOFs has been also studied by Zheng et al. [95]. Uptake of strontium and technetium with metal–organic frameworks has been also reported [96,97].

### 5.8. Extraction of Selenium

Selenium(IV) and selenium(VI) have been extracted from agricultural samples prior to their determination by electrothermal AAS with a nanocomposite consisting of MIL-101(Cr) and magnetite nanoparticles modified with dithiocarbamate [89]. The sorbent was found to be stable at acidic conditions since a solution of 0.064 mol L^−1^ HCl was chosen for elution and reusability for up to 12 times was reported. The herein developed method was successfully applied to water and agricultural samples for the determination of total selenium.

### 5.9. Multielement Extraction

HKUST-1 (MOF-199) material was used for the preconcentration of Cd(II) and Pb(II) ions from fish, sediment, and water samples prior to their determination by FAAS [90]. Trimesic acid and copper nitrate trihydrate were used for the synthesis of the material, which was further functionalized with Fe_3_O_4_@Py nanoparticles. Modification with pyridine was performed to increase selectivity toward the examined metal ions. The researchers came to the same conclusion regarding the material decomposition with hydrochloric acid and nitric acid. Therefore, elution was performed with 0.01 mol L^−1^ NaOH in EDTA solution. High adsorption capacity, low limit of detection, and high enrichment factor were achieved with the proposed MSPE sample preparation method.

HKUST-1 have been also employed for the extraction of Cd(II), Pb(II), and Ni(II) ions from seafood (fish and shrimps) and agricultural samples after modification with magnetic nanoparticles carrying covalently immobilized 4-(thiazolylazo) resorcinol (Fe_3_O_4_@TAR) [91]. TAR was utilized in this work as a chelator to show more selectivity toward the target analytes. Nitric acid was used for the acidic digestion of the samples and FAAS was used for the determination of the analytes. The adsorption and desorption steps were optimized with Box–Behnken experimental design [79]. Since HKUST-1 is not stable at acidic solutions, elution with EDTA was performed. The developed MSPE method was simple, selective, rapid, reproducible, and able to provide low LOD values and good extraction recoveries.

A magnetic copper benzene-1,3,5-tricarboxylate metal–organic framework functionalized with Fe_3_O_4_-benzoyl isothiocyanate nanoparticles was employed for the MSPE of Cd(II), Pb(II), Zn(II), and Cr(III) from vegetable samples prior to their determination by FAAS [54]. Modification with benzoyl isothiocyanate was performed to increase the selectivity toward the examined metals. Box–Behnken experimental design in combination with response surface methodology was used for the optimization of the adsorption and desorption steps [79]. The MSPE method was successfully used for the analysis of leek, parsley, fenugreek, beetroot leaves, garden cress, coriander, and basil. For the elution step, decomposition of the MOF was observed with hydrochloric acid, nitric acid, and sodium hydroxide, while EDTA and thiourea provided satisfactory recoveries without structure decomposition. Compared with Fe_3_O_4_-benzoyl isothiocyanate sorbent, the developed MOF material exhibited higher extraction efficiency. The novel method was simple and rapid while it provided good extraction efficiency and high enhancement factors.

The same elements have been extracted from agricultural samples with MIL-101(Fe) functionalized with Fe_3_O_4_-ethylenediamine prior to their determination by FAAS [92]. The presence of ethylenediamine in the sorbent enhances the selectivity of the sorbent toward the reported metals. Adsorption and desorption steps were optimized with Box–Behnken experimental design and response surface methodology [79]. MIL-101(Fe) was synthesized by iron chloride hexahydrate and terephthalic acid. Elution with EDTA was performed to avoid decomposition of the material. Leek, fenugreek, parsley, radish, radish leaves, beetroot eaves, garden cress, basil, and coriander were successfully analyzed with the developed MSPE method. Trace amounts of metal ions can be determined in a relatively high volume of samples due to the high preconcentration factor of the MSPE procedure.

A copper-(benzene-1,3,5-tricarboxylate) MOF material functionalized with dithizone-modified Fe_3_O_4_ nanoparticles (Fe_3_O_4_@DHz) and used for the preconcentration of Cd(II), Pb(II), Ni(II), and Zn(II) ions [60]. The modification with dithizone enhances the selectivity toward the examined metals. For the synthesis of the MOF material, trimeric acid in DMF/ethanol (1:1 v/v) was mixed with an ethanol solution of Fe_3_O_4_@DHz and copper acetate monohydrate and the mixture was heated at 70 °C under stirring for 4 h. Box–Behnken design through response surface methodology was used for the extraction optimization and FAAS was implemented for the detection of the analytes [79]. Elution of the adsorbed analytes was performed with 0.01 mol L^−1^ NaOH in thiourea to avoid any structure decomposition. The developed method provided low LODs, good recovery values, and high enhancement factors for the examined heavy metal ions.

Lanthanide Metal–Organic Frameworks have been also evaluated for their suitability as sorbents for the adsorption of heavy metal ions. In 2016, Jamali et al. [55] synthesized MOF materials using terbium hexahydrate, dysprosium nitrate hexahydrate, erbium nitrate hexahydrate, and ytterbium nitrate hexahydrate with trimesic acid in DMF. The reaction mixture was heated into a Teflon-lined reactor at 105 °C for 24 h. The novel dysprosium MOF exhibited high surface area as well as high dispersibility in aqueous solutions and it was found to be the most selective among the four examined material and it was further employed for the d-SPE of Pb(II) and Cu(II) ions from environmental water samples prior to FAAS analysis. Elution of the adsorbed analytes was performed with 0.1 mol L^−1^ HNO_3_. The MOF material was found to be stable after the desorption process and it could be used for at least 5 times without loss of functionality. The developed method showed low LODs, good linearity, selectivity, and satisfactory recovery values.

Mechanosynthesized azine decorated zinc(II) organic frameworks have been evaluated for the extraction of Cd(II), Co(II), Cr(III), Cu(II), and Pb(II) from water samples prior to their determination by flow injection ICP-OES [24]. The TMU-4, TMU-5, and TMU-6 examined metal–organic frameworks were prepared by the mechanochemical of zinc acetate dihydrate, 4,4′-oxybisbenzoic acid and an N-donor ligand. The ligands were 1,4-bis(4-pyridyl)-2,3-diaza1,3-butadiene, 2,5-bis(4-pyridyl)-3,4-diaza-2,4-hexadiene(4-bpmb), and N1,N4-bis((pyridin-4-yl)methylene)-benzene-1,4-diamine for TMU-4, TMU-5, and TMU-6, respectively. The novel sorbents were stable in water and a wide range of pH values while they provided high adsorption capacity. A solution of 0.4 mol L^−1^ EDTA was used for the elution of the analytes. It was indicated that for trace amounts of heavy metals, the basicity of the N-donor ligands in the groups of the MOF material is critical for the adsorption efficiency, while for high concentrations of metal ions the main factor that influences the adsorption process is the void space of the MOFs.

Safari et al. [93] prepared metal–organic frameworks with and without modification with azine groups and used them for the MSPE of Co(II), Cu(II), Pb(II), Cd(II), Ni(II), Cr(III), and Mn(II). For this purpose, TMU-8 and TMU-9 metal–organic frameworks were prepared from cadmium nitrate tetrahydrate, 4,4′-oxybisbenzoic acid, and a ligand. TMU-8 contained 1,4-bis(4-pyridyl)-2,3-diaza-1,3-butadiene as a ligand, while TMU-9 contained 4,4′-bipyridine. It was found that the azine-containing TMU-8 showed better adsorption capability compared to TMU-9 that did not have azine groups. Finally, magnetic TMU-8 was prepared by the in-situ synthesis of a magnetic core-shell nanocomposite. Adsorption and desorption steps were optimized with central composite design (CCD) in combination with a Bayesian regularized artificial neural network technique. The novel sorbent was prepared from a cadmium complex compound, 4,4′-oxybisbenzoic acid, and a ligand and it was successfully applied for the analysis of environmental water samples prior to ICP-OES detection. Elution of the analytes was performed with 0.5 mol L^−1^ HNO_3_, however, no data about material stability or sorbent reusability were provided.

Wu et al. [94] used a crystalline highly porous copper terephthalate MOF for the sample preparation of samples containing heavy metal ions after its post-synthetic modification. The MOF material was prepared from copper nitrate trihydrate and terephthalic acid in DMF with at 100 °C for 24 h. The material was dispersed dehydrated alcohol and the thiol-functionalized copper terephthalate nanoparticles were obtained with the addition of dithioglycol after stirring at room temperature for 24 h. The novel sorbent was used for the extraction of four heavy metals Hg(II), Cr(VI), Pb(II), and Cd(II) showing remarkable extraction efficiency, especially for mercury. EDTA was used to desorb the analytes. However, the addition of EDTA caused a structural collapse to the sorbent, which limited its reusability to up to three times. The d-SPE method was successfully applied for the preconcentration of the metal ions from tea samples prior to their determination with AFS (for mercury) and AAS (for chromium, lead and cadmium).

### 5.10. Application of ZIFs for the Extraction of Metal Ions

Zeolitic imidazolate frameworks are a subclass of metal–organic frameworks structured with Zn(II) or Co(II) ions and imidazolate and its derivatives, combining the benefits of zeolites and MOFs [98,99]. In 2016, Zou et al. [100] used a magnetic ZIF-8 material for the ultrasensitive determination of inorganic arsenic by hydride generation-atomic fluorescence spectrometry. ZIF-8 was synthesized from zinc nitrate hexahydrate and 2-methylimidazole. The obtained nanoparticles were functionalized with Fe_3_O_4_. The adsorption of arsenic took place in 6 h, following by dissolution of the sorbent in hydrochloric acid to assist the desorption procedure. The novel MSPE method was successfully employed for the extraction of inorganic arsenic from water and urine samples. It has been reported that unlike other metal–organic frameworks, ZIF-8 has exceptional thermal and chemical stability in water and aqueous alkaline solutions, which makes it an appropriate sorbent for sample preparation [101]. However, in the hydrochloric acid solution, the sorbent was completely dissolved, indicating low stability in acidic solution and no potential sorbent reusability [100].

### 5.11. Application of COFs for the Extraction of Metal Ions

Covalent organic frameworks (COFs) are structurally related materials with MOFs that consist of light elements (H, O, C, N, B, Si) connected with organic monomers through strong covalent bonds [102,103]. COFs are a novel type of ordered crystalline porous polymers that exhibit superior properties such as low crystal density, high specific surface area, tunable pore size, and very good thermal stability [104]. In 2018, Liu et al. [105] fabricated porous covalent organic frameworks and used them as a selective advanced adsorbent for the on-line preconcentration of trace elements against from complex sample matrices. For this purpose, two different COF materials were synthesized. The first COF was prepared from 1,3,5-triformylphloroglucinol and benzidine. For the preparation of the second COF, 1,3,5-triformylphloroglucinol was functionalized with diglycolic anhydride to decorate the carboxylic groups. Accordingly, the functionalized 1,3,5-triformylphloroglucinol was mixed with benzidine 1,4-dioxane and mesitylene. The two COF materials were packed into cartridges and were employed for the on-line solid-phase extraction of Cr (III), Mn (II), Co (II), Ni (II), Cd (II), V (V), Cu (II), As (III), Se (IV), and Mo (VI) prior to their determination by ICP-MS. Due to the presence of the carboxylic groups, the second COF showed effective adsorption behavior for more than 10 metal ions, while the non-functionalized COF showed effective adsorption behavior for only five metal ions. The porous COFs exhibited superior chemical and thermal stability as well as a large surface area. The developed method was successfully applied for the analysis of milk and wastewater samples.

## 6. Conclusions

For the use of metal–organic frameworks in the field of analytical chemistry, we conclude that they offer a further interesting possibility by enriching the analytical toolbox for trace metal analysis. One advantage of MOFs is their high surface area, which leads to high extraction efficiency and enrichment factors. Compared with other sorbent materials (including activated carbon and graphite-based materials), MOFs have also the advantage of tunable and homogeneous pores of specific sizes. However, until now there are only a few research articles regarding the extraction of metal ions with MOFs prior to their determination by a spectrometric technique.

On the other hand, a significant disadvantage of various MOFs is their instability in aqueous solution. In contrast with the environmental remediation applications of MOFs in which the researchers focus on the stability of the material only under adsorption conditions, in analytical chemistry quantitative desorption of the metal ion is essential in order to provide satisfactory recovery, no carry-over effect, and satisfactory sorbent reusability. Even though most of the studied MOFs were found to be stable under adsorption conditions at intermediate pH values, acidic desorption was found to cause their structure decomposition. In order to overcome this problem, milder eluents including EDTA, NaCl, and NaOH in EDTA or thiourea were evaluated. However, only a few sorbents were found to be reusable after the desorption step, which is considered a significant drawback for MOF sorbents.

Recent advances in the preparation of MOFs include chemical pre- or post-synthetic modification and functionalization in order to overcome their well-known limitation of water instability, which reduce their possible application to real sample analysis.

Moreover, the selectivity of MOFs toward specific metal ions is considered relatively low. This limitation can be overcome with functionalization of metal–organic frameworks with compounds like dithizone that can form chelating complex compounds with the target analytes and extract metal ions through chelation.

Until today, MOFs have been used for the extraction of metal ions by a limited number of extraction techniques. Future applications of MOFs as sorbents in other extraction formats such as SBSE or on-line techniques should be investigated. Since only a limited amount of metal ions have been extracted with MOFs, in-depth study using extraction formats such as SPE, MSPE, d-SPE, and PT-SPE also need to be performed. Finally, metal–organic frameworks have to be evaluated for the sample preparation of more sample matrices including agricultural, biological, environmental, and food samples.

## Figures and Tables

**Table 1:** Applications of metal–organic frameworks for the extraction of metal ions.

Analyte	Organic Linker of MOF	Metal of MOF	Modification	Matrix	Sample Preparation Technique	Detection Technique	Recovery (%)	LOD (ng mL^−1^)	Reusability	Ref.
Pd(II)	Trimesic acid	Cu	Fe_3_O_4_@Py	Fish, sediment, soil, water,	MSPE	FAAS	96.8–102.5	0.37	-	[80]
	Malonic acid	Ag	-	Water	SPE	FAAS	>95	0.5	Up to 5 times	[65]
Pb(II)	Trimesic Acid	Cu	DHz, Fe_3_O_4_	Water	MSPE	ETAAS	97–102	0.0046	At least 80 times	[81]
	Trimesic Acid	Cu	Fe_3_O_4_@SH	Rice, pig liver, tea, water	MSPE	FAAS	>95	0.29–0.97	-	[77]
	meso-tetra(4-carboxyphenyl) porphyrin	Zr	-	Cereal, beverage, water	d-SPE	FAAS	90–107	1.78	Up to 42 times	[82]
	Trimesic acid	Cu	Fe_3_O_4_@4-(5)-imidazole-dithiocarboxylic acid	Fish, canned tune	MSPE	CVAAS	95–102	10	At least 12 times	[83]
Hg(II)	Trimesic acid	Cu	Thiol-modified silica	Fish, sediment, water	d-SPE	CV-AAS	91–102	0.02	-	[78]
	3′5,5′-azobenzenetetracarboxylic acid	Cu	-	Tea, mushrooms	d-SPE	AFS	Average 93.3	>0.58 mg kg^−1^	Up to 3 times	[84]
	Benzoic acid and meso-tetrakis(4-Carboxyphenyl)porphyrin	Zr	-	Fish	PT-SPE	CVAAS	74.3–98.7	20 × 10^−3^	At least 15 times	[76]
Cu (II)	Aminoterephthalic acid	Zn	Fe_3_O_4_	Water	MSPE	ETAAS	98–102	0.073		[29]
Cd(II)	Terephthalic acid	Fe	Fe_3_O_4_@MAA, AMSA	Water	MSPE	FAAS	>96	0.04	Up to 10 times	[85]
Th(IV)	2 –hydroxyterephthalic acid	Zr	-	Water	d-SPE	Spectrophotometry	>90	0.35	At least 25 times	[86]
	[1,1′-biphenyl]-4-carboxylic acid	Eu	-	Water	Probe	UV	N.A.	24.2	N.A.	[87]
U(VI)	4,4′,4″-(1,3,5-triazine-2,4,6-triyltriimino)tris-benzoic acid	Te	-	Water	d-SPE	ICP-MS	94.2–98.0	0.9	At least 3 times	[88]
Se(IV), Se(VI)	Terephthalic acid	Cr	Fe_3_O_4_@dithiocarbamate	Water, agricultural samples	MSPE	ETAAS	>92	0.01	Up to 12 times	[89]
Cd(II), Pb(II)	Trimesic acid	Cu	Fe_3_O_4_@Py	Fish, sediment water	MSPE	FAAS	92.0–103.3	0.2–1.1	-	[90]
Cd(II) Pb(II) Ni(II)	Trimesic acid	Cu	Fe_3_O_4_@TAR	Sea food, agricultural samples	MSPE	FAAS	83–112	0.15–0.8	-	[91]
Cd(II), Pb(II), Zn(II) Cr(III)	Trimesic acid	Cu	Fe_3_O_4_-benzoyl isothiocyanate	Vegetables	MSPE	FAAS	80–114	0.12–0.7	-	[54]
	Terephthalic acid	Fe	Fe_3_O_4_-ethylenediamine	Agricultural samples	MSPE	FAAS	87.3–110	0.15–0.8	-	[92]
Cd(II), Pb(II), Ni(II), Zn(II)	Trimesic Acid	Cu	Fe_3_O_4_@DHz	Fish, sediment, soil, water	MSPE	FAAS	88–104	0.12–1.2	-	[60]
Pb(II), Cu(II)	Trimesic acid	Dy	-	Water	d-SPE	FAAS	95–105	0.26–0.40	At least 5 times	[55]
Cd(II), Co(II), Cr(III), Cu(II), Pb(II)	4-bpmb	Zn	-	Water	d-SPE	ICP-OES	90–110	0.01–1	-	[24]
Co(II), Cu(II), Pb(II), Cd(II), Ni(II), Cr(III), Mn(II)	4,4′-oxybisbenzoic acid	Cd	Fe_3_O_4_	Water	MSPE	ICP-OES	>90	0.3–1	-	[93]
Hg(II), Cr(VI) Pb(II) Cd(II)	Terephthalic acid	Cu	Dithioglycol	Tea	d-SPE	AFS, AAS	95–99	Not mentioned	Up to 3 times	[94]
